# Utilization, Safety, and Technical Performance of a Telemedicine System for Prehospital Emergency Care: Observational Study

**DOI:** 10.2196/14907

**Published:** 2019-10-08

**Authors:** Marc Felzen, Stefan Kurt Beckers, Felix Kork, Frederik Hirsch, Sebastian Bergrath, Anja Sommer, Jörg Christian Brokmann, Michael Czaplik, Rolf Rossaint

**Affiliations:** 1 Department of Anesthesiology Medical Faculty RWTH Aachen University Aachen Germany; 2 Emergency Department Maria Hilf-Hospital Moenchengladbach Moenchengladbach Germany; 3 Department of Health, Ethics and Society Care and Public Health Research Institute Maastricht University Maastricht Netherlands; 4 Emergency Department Medical Faculty RWTH Aachen University Aachen Germany

**Keywords:** emergency medicine, ambulances, telemedicine, quality of care, eHealth

## Abstract

**Background:**

As a consequence of increasing emergency medical service (EMS) missions requiring an EMS physician on site, we had implemented a unique prehospital telemedical emergency service as a new structural component to the conventional physician-based EMS in Germany.

**Objective:**

We sought to assess the utilization, safety, and technical performance of this telemedical emergency service.

**Methods:**

We conducted a retrospective analysis of all primary emergency missions with telemedical consultation of an EMS physician in the City of Aachen (250,000 inhabitants) during the first 3 operational years of our tele-EMS system. Main outcome measures were the number of teleconsultations, number of complications, and number of transmission malfunctions during teleconsultations.

**Results:**

The data of 6265 patients were analyzed. The number of teleconsultations increased during the run-in period of four quarters toward full routine operation from 152 to 420 missions per quarter. When fully operational, around the clock, and providing teleconsultations to 11 mobile ambulances, the number of teleconsultations further increased by 25.9 per quarter (95% CI 9.1-42.6; *P*=.009). Only 6 of 6265 patients (0.10%; 95% CI 0.04%-0.21%) experienced adverse events, all of them not inherent in the system of teleconsultations. Technical malfunctions of single transmission components occurred from as low as 0.3% (95% CI 0.2%-0.5%) during two-way voice communications to as high as 1.9% (95% CI 1.6%-2.3%) during real-time vital data transmissions. Complete system failures occurred in only 0.3% (95% CI 0.2%-0.6%) of all teleconsultations.

**Conclusions:**

The Aachen prehospital EMS is a frequently used, safe, and technically reliable system to provide medical care for emergency patients without an EMS physician physically present. Noninferiority of the tele-EMS physician compared with an on-site EMS physician needs to be demonstrated in a randomized trial.

## Introduction

### Background

German emergency medical services (EMSs) comprise a system of cooperating on-site paramedics and physicians who are separately dispatched to the patient depending on the severity of the emergency. Over recent years, EMSs have faced a serious problem: the number of missions requiring on-site EMS physicians has continuously been rising [1], making dispatchable EMS physicians a scarce resource.

Several factors have facilitated this development. First, by German law, only physicians are entitled to prescribe drugs. Paramedics are allowed to administer drugs only if (1) the situation is life-threatening, (2) the administration and dosing are predefined in a standard operating procedure (SOP), (3) a less invasive measure to achieve the same effect is not available, and (4) an EMS physician has already been dispatched to the scene. Hence, EMS physicians are regularly dispatched to patients with non–life-threatening conditions (eg, being in pain) and are—during that time—indispensable for other potentially more severe emergency missions. Second, in one-fifth of emergency missions involving an EMS physician, the EMS physician is subsequently requested by the paramedics on site and was not dispatched initially [2]. As a consequence, the treatment of these patients is distinctly delayed. Third, overall mission numbers have continuously been increasing with a stable proportion of around 45%, requiring an EMS physician on site [3]. This has led to a higher workload and longer arrival times [4], and moreover, this trend could not be reversed, despite a nationwide increase in EMS physician stations.

### Objectives

To overcome these shortcomings, we developed a holistic prehospital telemedical emergency service for the City of Aachen. The routine EMS, including separately dispatchable paramedics and physicians, was complemented with an additional tele-EMS physician [5,6]. During emergency missions for which only paramedics were dispatched, these paramedics can—if needed—either request an on-site EMS physician to be dispatched to the scene or consult with the tele-EMS physician for the treatment of the patient. On consultation, the tele-EMS physician communicates via voice and has immediate access to the vital data of the patient and the Global Positioning System (GPS) coordinates of the ambulance. Paramedics can instantaneously send pictures to the tele-EMS physician, and video can be streamed from the inside of the ambulance. This setting allows for the telemedical delegation (teledelegation) of predefined medications including opioids and has been demonstrated to provide patients with standard care in accordance with treatment guidelines and without complications [7-9]. After initial establishment and evaluation [6,10], the system has been integrated as an around-the-clock routine component of the City of Aachen EMS. In this retrospective analysis of the first 3 operational years, we sought to assess the utilization, safety, and technical performance of the holistic Aachen prehospital telemedical emergency service.

## Methods

### Patients

All patients in primary emergency missions with consultation of the tele-EMS physician during the first 3 operational years (April 2014 to March 2017) were included in the analyses. Tele-EMS physicians operate from a tele-EMS control center in close proximity to the mission control center of the EMSs. Tele-EMS physicians communicate with especially equipped ambulances via voice and have immediate access to the vital data of the patient and the GPS coordinates of the ambulance. In addition, video can be streamed from the inside of the ambulance. After making a working diagnosis, tele-EMS physicians are prompted with a guideline-based SOP, including corresponding treatment algorithms and corresponding checklist. Previous publications provide a detailed description of the establishment and functionality as well as technical details of the Aachen tele-EMS physician [5-7,11].

### Data Sources and Analyses

#### Mission and Patient Data

Age, sex, National Advisory Committee for Aeronautics (NACA) scores, categorized main symptoms, administration of nonopioid drugs and opioid drugs, and teleconsultation times were abstracted from the protocols archived by the tele-EMS physician. Transport modalities and mission times of involved mobile EMS forces (paramedics and on-site EMS physician) were abstracted from the control system of the dispatch center (COBRA 4; ISE).

#### Safety

Adverse events were assessed in a 2-step process. First, we preselected protocols if (1) the free-text comments section contained parts of or the German words or word combinations for unsuccessful, instable, unstable, no sign of recovery, on-site physician, hypotensive, hypotension, allergy, allergic, anaphylaxis, anaphylactic, accident, accidental, erroneous, error, confusion, confused, and possible misspellings; or (2) the tele-EMS physician administered catecholamines (adrenaline, noradrenaline, and theodrenaline-cafedrine), antihistamines, or corticosteroids. Second, these protocols were reviewed by two independent researchers, and disagreements were resolved by consensus.

#### Technical Performance

During these first 3 operational years, tele-EMS physicians were asked to regularly fill out a paper-based questionnaire after each teleconsultation to assess the technical performance of the system. *Transmission quality of the telemetric components*, *two-way voice communication*, *GPS coordinates*, *real-time vital data*, *12-lead resting* *electrocardiogram (ECG)*, *still pictures*, and *one-way video stream* were assessed in mutually exclusive categories *no malfunctions*; *some malfunctions, quality not affected*; *malfunctions, quality reduced*; and *malfunctions, transmission impossible*. Teleconsultations with impossible transmission of voice communication, real-time vital data, 12-lead resting ECG, still pictures, and video stream were considered complete system failures.

#### Statistical Analyses

Frequencies are reported as proportion and percentage and numerical values as median and interquartile range (IQR), without prior testing for normal distribution. Trends were fitted using univariable linear regression. Slopes were reported with 95% CI and whether the slope differed significantly from 0. CIs for the proportion of a count were analyzed using the method described by Wilson [12]. Linear regression modeling was conducted, and figures were created with Prism version 8.0.2 (GraphPad) for Mac operating system (OS). All other analyses were conducted with RStudio version 1.1.463 for Mac OS (RStudio) operating R version 3.5.2 for Mac OS. A type I error of 5% or less was considered statistically significant.

### Patient and Public Involvement

Patients and the public were not involved in the design, conduct, and reporting of the research.

### Ethics Approval and Consent to Participate

The local ethics committee granted analysis of the data for quality assurance purposes and waived the requirement of informed consent (EK109/15, University Hospital RWTH Aachen).

### Availability of Data and Material

The datasets used and/or analyzed during this study are available from the corresponding author on reasonable request.

## Results

### Patients, Mission, and Patient data

During the study period, tele-EMS physicians were consulted in primary emergency missions for 6265 patients. Patients had a median age of 70 years (IQR 48-81), and 52.88% (3313/6265) were female. Of the 6265 patients, most were categorized as NACA III (3594/6265, 57.36%) and NACA IV (1445/6265, 23.06%) cases. A total of 6.43% presented a life-threatening condition (403/6265), indicated by an NACA score of V or greater. The majority of patients (4328/6265, 69.08%) were treated on-site by the tele-EMS physician and subsequently transferred to a hospital, as little as 7.80% (489/6265) were neither treated on-site nor transferred to the hospital (Table 1).

**Table 1:** Characteristics of the patients treated in 6265 teleconsultations in primary emergency missions by tele-emergency medical service physicians of the Aachen telemedical prehospital emergency service.

Characteristics		Values
Age (years), median (IQR)^a,b^		70 (48-81)
**Sex, n (%)^c^**		
	Female	3313 (52.88)
	Male	2844 (45.40)
**Severity of the emergency, n (%)^d^**		
	NACA^e^ I	47 (0.75)
	NACA II	457 (7.29)
	NACA III	3594 (57.37)
	NACA IV	1445 (23.06)
	NACA V	394 (6.29)
	NACA VI	3 (0.05)
	NACA VII	8 (0.13)
**Type of main symptom, n (%)**		
	Circulatory	1146 (18.29)
	Neurologic	1049 (16.74)
	Cardiac	852 (13.60)
	Trauma	600 (9.58)
	Abdominal	536 (8.56)
	Other	2082 (33.23)
**Mission details, n (%)^f^**		
	On-site treatment and transfer to the hospital	4326 (69.05)
	Transfer to the hospital only and no on-site treatment	1438 (22.95)
	On-site treatment only and no transfer to the hospital	338 (5.40)
	No on-site treatment and no transfer to the hospital	489 (7.81)

^a^IQR: interquartile range.

^b^Data of 232 cases missing.

^c^Data of 107 cases missing.

^d^Data of 317 cases missing

^e^NACA: National Advisory Committee for Aeronautics.

^f^Do not add up to 100% because categories are not mutually exclusive.

### Utilization of the System

Teleconsultations increased during the first 4 quarters of routine operations from 152 to 420, as subsequently more ambulances became technically equipped for consultations with the tele-EMS physician (Multimedia Appendix 1). On successful implementation of an around-the-clock tele-EMS physician service for 11 ambulances from the second quarter of 2015, teleconsultations further increased by 25.9 per quarter (95% CI 9.1-42.6; *P*=.009). In total, the tele-EMS physician delegated the application of nonopioid drugs in 81.5% (5111/6265; 95% CI 80.6%-82.5%) and opioid drugs in 20.7% (1297/6265; 95% CI 19.7%-21.7%) of the teleconsultations (Multimedia Appendix 2). The teledelegated application of nonopioids increased similarly with increasing mission numbers (17.3 per quarter; 95% CI 3.1-31.4; *P*=.02), as did the teledelegated application of opioids (12.0 per quarter; 95% CI 0.8-23.3; *P*=.04). In fact, when analyzing all emergency missions involving any EMS physician in the city of Aachen, the proportion of missions carried out with the newly established tele-EMS physician continuously increased, whereas the proportion of missions carried out with a conventional on-site EMS physician continuously decreased (0.9% per quarter; 95% CI 0.3%-1.4%; *P*=.08; Multimedia Appendix 1).

Overall, 91.10% of the teleconsultations (5708/6265; 95% CI 90.3%-91.8%) were used to support the on-site paramedics. In 3.65% of the teleconsultations (229/6265; 95% CI 3.2%-4.2%), the tele-EMS physician saw the need to dispatch an on-site EMS physician. On-site paramedics consulted with the tele-EMS physician in 2.45% (154/6265; 95% CI 2.1%-2.9%) of the missions to bridge the time until a simultaneously dispatched EMS physician arrived on the scene. The tele-EMS was contacted for support after an on-site physician already had arrived on site in 4.38% (275/6265; 95% CI 3.9%-4.9%) of all teleconsultations.

### Safety

Only 6 of 6265 patients (0.09%; 95% CI 0.04%-0.21%) experienced adverse events. One patient became unconscious after being treated with teledelegated nitroglycerine for acute coronary syndrome, an on-site physician was immediately dispatched to the scene by the tele-EMS physician, and the patient regained consciousness after 10 seconds and presented stable vital signs and full orientation until arrival at the hospital. A 94-year-old woman entered a highly agitated state after receiving ketamine for the treatment of a hip fracture. An on-site physician was dispatched, and the patient was sedated with midazolam and safely transferred to the hospital. One patient received reproterol (Bronchospasmin, a beta-2-mimetic) instead of scopolamine (Buscopan, a parasympatholytic) because of the phonetical similarity of the German trade names; however, the patient did not suffer from any side effects. Paramedics accidentally punctured the cubital artery instead of a vein, which was instantaneously recognized and adequately treated with a pressure bandage. In addition, 2 patients suffered from erythema: 1 patient presented a generalized erythema and pruritus to the application of morphine (sufficiently treated with histamine antagonists), and the other presented a local erythema after the infusion of metamizole (the infusion was stopped, and no signs of generalized anaphylactic reactions were observed). All patients with adverse events presented normal vital data at arrival in the emergency department.

### Technical Performance

Tele-EMS physicians assessed the technical performance of the system by filling out the paper-based questionnaire following 5220 of all 6265 teleconsultations (83.32%). Two-way voice communication was used in all 5220 (100.00%) assessed teleconsultations. Real-time vital data were transmitted in 95.57% (4989/5220), GPS coordinates in 92.66% (4837/5220), 12-lead resting ECGs in 47.26% (2467/5220), still pictures in 45.84% (2393/5220), and one-way video streams in 44.25% (2310/5220) of consultations with the tele-EMS physician.

**Figure 1:**
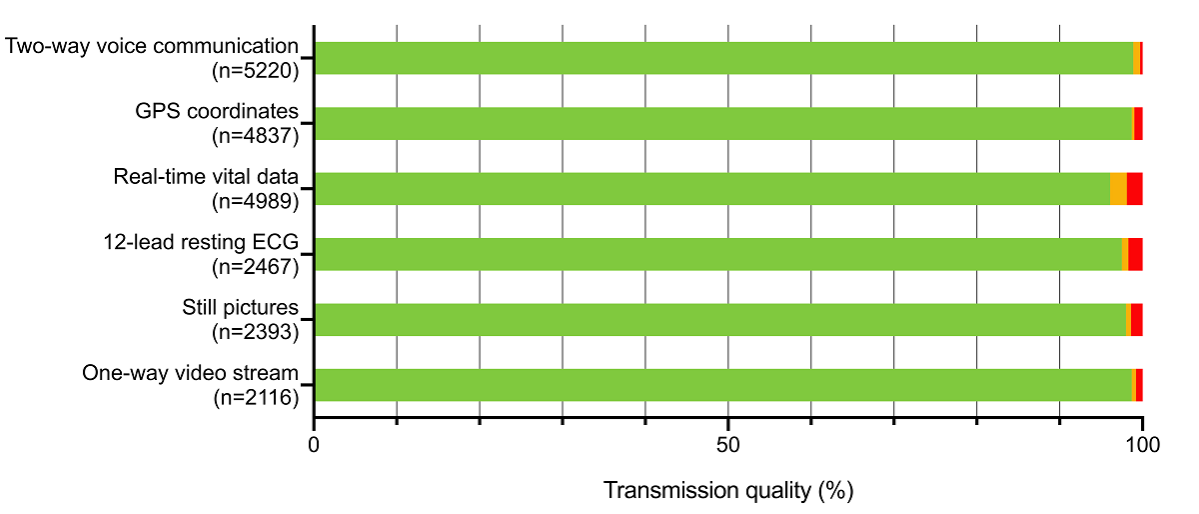
Technical performance of the Aachen physician-staffed telemedical prehospital emergency service in 5220 of 6265 teleconsultations by transmission component and quality: transmission quality not affected (green), transmissions with reduced quality (yellow), and complete transmission component malfunction (red). ECG: electrocardiogram; GPS: Global Positioning System.

Complete malfunctions of single transmission components with no possible transmission occurred from as low as 0.26% (14/5220; 95% CI 0.2%-0.5%) during two-way voice communications, more than 1.01% (53/5220; 95% CI 0.8%-1.3%) during GPS coordinate transmissions, 1.34% (70/5220; 95% CI 1.1%-1.7%) during one-way video stream transmissions, 1.43% (72/5220; 95% CI 1.1%-1.7%) during still picture transmissions, 1.66% (87/5220; 95% CI 1.3%-2.1%) during 12-lead resting ECG transmissions to as high as 1.91% (100/5220; 95% CI 1.6%-2.3%) during real-time vital data transmissions (Figure 1). Complete system failures occurred in only 0.34% (18/5220; 95% CI 0.2%-0.6%) of all teleconsultations.

## Discussion

### Principal Findings

In response to the increasing mission numbers involving on-site physicians in German EMSs, we had developed a holistic prehospital telemedical emergency service for the City of Aachen, which began routine operations in 2014. In this retrospective analysis of the first 3 operational years, we sought to assess the utilization, safety, and technical performance of the system. We found that paramedics consulted the tele-EMS physician on a regular basis in 6265 primary emergency missions with a continuing increase of 26 teleconsultations per quarter. Of all missions involving any EMS physician, the proportion of missions carried out with the tele-EMS physician increased by 4% per year. Telemedical treatment by the tele-EMS physician resulted in as little as 6 complications in 6265 teleconsultations (0.1%). In a convenience sample assessing the technical performance of the system, we found that single transmission components malfunctioned within a range of 0.3% (two-way voice communication) to 1.9% (real-time vital data stream) in 5220 teleconsultations. Complete system failures occurred very rarely in only 18 teleconsultations (0.3%).

### Strengths and Weaknesses and Comparison With Other Studies

This is the world’s largest analysis of cases that were provided with routine medical care within a holistic telemedical system for prehospital emergency care. We were able to analyze all teleconsultations for primary emergency missions during the first 3 operational years of the system. The fact that we did not analyze a sample combined with the high number of cases contributes to the internal validity of our results. On the other hand, the uniqueness of the Aachen prehospital telemedical emergency service limits the generalizability to other (even similar) systems.

The steep increase in teleconsultations during the first operational year has to be ascribed primarily to the increasing number of ambulances (from 2 to 11) being subsequently equipped for telemedical operations. In addition, the tele-EMS service expanded its availability from a 12-hour day to an around-the-clock service during the first year. However, during the 2-year routine service with an around-the-clock tele-EMS physician and 11 operational ambulances, the number of teleconsultations further increased, as did the teledelegation of nonopioid drugs and opioids. This is most likely because of a training effect operating the new telemedical emergency care system. Paramedics may have overcome barriers in applying the new system more easily [13] and may have recognized increasing possibilities to work with the new system instead of requesting a *traditional* on-site EMS physician [14,15].

The number of complications in our analyses was low. This may have been because of the fact that we analyzed routine treatment protocols and that complications or adverse events may not have been documented. Complications such as the occurrence of common side effects (nausea after the administration of opioids and relevant hypotension after the administration of metamizole), mild allergic reactions, or confusion of phonetically similar drugs (eg, midazolam and metamizole) may have occurred but not explicitly mentioned as they presumably could also have occurred with EMS physician present on site. Moreover, as all our tele-EMS physicians are anesthesiologists (board certified for anesthesiology and prehospital emergency care, longstanding experience in more than 500 on-site prehospital emergency missions, and certified provider of advanced life support and prehospital trauma life support), they could have easily considered these events as typical and treatable. In addition, none of the recorded complications were inherent in the system and could have easily happened exactly similar to this with an EMS physician treating the patient on-site. Still, a low complication rate concurs with other emergency telemedicine studies that have demonstrated high safety in comparable *nonserious* emergency patient populations groups [16,17] but also for patients with life-threatening conditions as, for example, ST-elevation myocardial infarction [18].

High rates of successful data transmission in our analysis concur with or outperform other studies that demonstrated telemedical data transmissions from mobile emergency units to be safe and reliable [11,19-21]. Being the most redundantly secured transmission component (headset from the Global System for Mobile Communications–equipped vital sign monitor, backup mobile phone, and any landline can be used), it comes as no surprise that two-way voice communication was least affected by transmission malfunctions. In our sample, a complete system failure without possible transmission occurred in only 0.3% of the teleconsultations. These events are highly likely caused by bad mobile service reception and can often easily be solved by relocating the transmission unit, that is, the ambulance. However, we are unable to determine whether technical problems were caused by malfunctions in the general telecommunication infrastructure or the telemedical service itself.

Unfortunately, the technical performance questionnaire was not filled out in 17% of the missions, leaving 83% of all teleconsultations to be analyzed. As this may very well have introduced bias to our study, this has most likely led to an overestimation of technical malfunctions, as tele-EMS physicians would rather fill out the report form after having experienced technical difficulties during the mission. In addition, we reviewed the mission protocols of the cases with complete transmission failures and found no irregularities, the missions were completed without adverse events, and the patients transferred to a hospital. The highest failure rate was detected when transmitting real-time vital data. This malfunction—and with the exception of 12-lead resting ECG and all other transmission components—can temporarily be overcome if the voice communication between the tele-EMS and the paramedics is still intact. In this manner, even the backup mobile phone’s automatic transmission of still pictures can be used as a fallback solution for failing 12-lead resting ECG transmissions.

Besides being frequently used, safe, and technically feasible, the holistic Aachen prehospital emergency system has been demonstrated to be effective, of high quality, and to provide instantaneous care to patients [22-26]: we have provided evidence in the past that the Aachen tele-EMS physician is as effective as conventional on-site EMS physicians when treating patients with pain [6,10], stroke [7], hypertensive crises [9], and acute coronary syndrome [8]. Similar effects have been provided by other work groups, as, for example, the prehospital telemedicine emergency care for patients with stroke leads to faster diagnosis and faster treatment compared with standard care [27,28]. These effects are most likely to be even more pronounced in rural areas where paramedics are noticeably faster available compared with on-site EMS physicians [29,30].

### Policy Implications and Conclusions

Life-threatening complications and errors can also occur in prehospital emergency care provided by an on-site physician [31]. During in-hospital life-threatening emergencies, it is possible and common for physicians to seek instantaneous support by more experienced colleagues. The on-site EMS physician does not have this possibility. Surprisingly, 275 teleconsultations in our study were made after an on-site physician arrived on the scene, presumably to provide support for the on-site EMS physician. The analyses of these cases will provide more insight into the effective application of our system for the purpose of on-site EMS physician support.

The increasing use of checklists in prehospital emergency care has fostered the adherence to standards and overall treatment quality [32]. The Aachen holistic prehospital telemedicine emergency service was designed to foster guideline-based treatment of patients. For this purpose, on entering a working diagnosis, the operating tele-EMS physician is automatically supplied with a guideline-based SOP, including treatment algorithms and corresponding checklists [6-9,11]. It is highly likely that the quality of care and safety of such a system is at least comparable with standard care provided by on-site EMS physicians for non–life-threatening emergencies. Therefore, this hypothesis is currently tested in a large randomized controlled trial [33].

In conclusion, this retrospective analysis of primary emergency care teleconsultations conducted during the first 3 operational years showed the Aachen prehospital EMS to be a frequently used, safe, and technically reliable system to provide medical care for prehospital emergency patients, without an EMS physician present. This telemedical emergency service is likely to provide high-quality emergency care for non–life-threatening events and could relieve mobile EMS physicians’ workload. Consequently, this may free EMS physicians for the treatment of more serious, life-threatening emergency missions and therefore provide faster high-quality care for all patients treated in EMSs.

## Appendix

Multimedia Appendix 1 Utilization of the Aachen prehospital telemedicine emergency system during the first 3 operational years. (A) Consultations of the tele-EMS physician for primary emergency missions (dark blue) increased strongly during the run-in phase (hatched gray), as did the teledelegated applications of nonopioid drugs (light blue) and opioids (orange). The number of the missions increased further after the tele-EMS physician became available around the clock for 11 ambulances at the beginning of the second quarter in 2015. (B) Of all primary emergency missions carried out including an EMS physician, the proportion of missions carried out with a tele-EMS physician increased by 4% (P < .05) after the run-in phase. EMS: emergency medical service.

Multimedia Appendix 2 Missions with on-site and tele-emergency medical service physicians in the City of Aachen during the first three operational years.

## Abbreviations

ECG: electrocardiogram
EMS: emergency medical service
GPS: Global Positioning System
IQR: interquartile range
NACA: National Advisory Committee for Aeronautics
OS: operating system
SOP: standard operating procedure
