# Supplementary figures and images for: Utilization, Safety, and Technical Performance of a Telemedicine System for Prehospital Emergency Care: Observational Study
Source: J Med Internet Res. 2019 Oct 8;21(10):e14907. doi: 10.2196/14907 (PMC6806125; doi:10.2196/14907)

**A**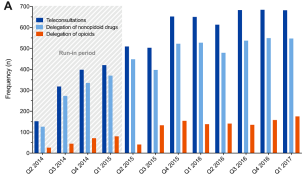**B**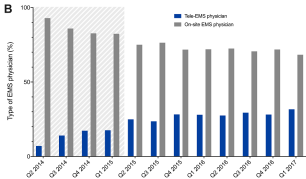

Supplement: Multimedia Appendix 1 [file jmir_v21i10e14907_app1.pdf]
