# Supplementary material for: Utilization, Safety, and Technical Performance of a Telemedicine System for Prehospital Emergency Care: Observational Study
Source: J Med Internet Res. 2019 Oct 8;21(10):e14907. doi: 10.2196/14907 (PMC6806125; doi:10.2196/14907)
Supplement: Multimedia Appendix 2 [file jmir_v21i10e14907_app2.pdf]

**Multimedia Supplement:** Missions with on-site and tele-EMS physicians in the City of Aachen during the first three operational years (left). The percentage of tele-EMS The right column depicts the teledelegation of non-opioids and opioids. These numbers are the basis for Figure 1.

| | Missions with (n = 27,027) | | Tele-EMS physician missions with teledelegation of | |
|---|---|---|---|---|
| | On-site EMS-physician[a] (n = 20,761) | Tele-EMS physician[a] (n = 6,265) | Non-opidoids[b] (n = 5,110) | Opidoids[b] (n = 1,296) |
| Q2 2014 | 2,000/2,152 (92,9%) | 152/2,152 (7,1%) | 126/152 (82.9%) | 26/152 (17.1%) |
| Q3 2014 | 1,941/2,259 (85,9%) | 318/2,259 (14,1%) | 273/318 (85.8%) | 45/318 (14.2%) |
| Q4 2014 | 1,904/2,302 (82,7%) | 398/2,302 (17,3%) | 335/398 (84.2%) | 71/398 (17.8%) |
| Q1 2015 | 1,966/2,386 (82,4%) | 420/2,386 (17,6%) | 370/420 (88.1%) | 80/420 (19.0%) |
| Q2 2015 | 1,530/2,039 (75,0%) | 509/2,039 (25,0%) | 448/509 88.0%) | 41/509 (8.1%) |
| Q3 2015 | 1,628/2,131 (76,4%) | 503/2,131 (23,6%) | 397/503 (78.9%) | 133/503 (26.4%) |
| Q4 2015 | 1,655/2,307 (71,7%) | 652/2,307 (28,3%) | 522/652 (80.1%) | 154/652 (23.6%) |
| Q1 2016 | 1,671/2,321 (72,0%) | 650/2,321 (28,0%) | 527/650 (81.1%) | 138/650 (21.2%) |
| Q2 2016 | 1,615/2,228 (72,5%) | 613/2,228 (27,5%) | 479/613 (78.1%) | 141/613 (23.0%) |
| Q3 2016 | 1,640/2,323 (70,6%) | 683/2,323 (29,4%) | 537/613 (87.6%) | 135/613 (22.0%) |
| Q4 2016 | 1,745/2,429 (71,8%) | 684/2,429 (28,2%) | 549/684 (80.3%) | 158/685 (23.1%) |
| Q1 2017 | 1,470/2,152 (68,3%) | 682/2,152 (31,7%) | 547/682 (80.2%) | 175/682 (25.7%) |

[a]percentages have been calculated as proportion of all missions during the respective quarter; [b]percentages have been calculated of all tele-EMS physician missions during the requested quarter.
